# GPER1 Silencing Suppresses the Proliferation, Migration, and Invasion of Gastric Cancer Cells by Inhibiting PI3K/AKT–Mediated EMT

**DOI:** 10.3389/fcell.2020.591239

**Published:** 2020-12-21

**Authors:** En Xu, Xuefeng Xia, Chaoyu Jiang, Zijian Li, Zhi Yang, Chang Zheng, Xingzhou Wang, Shangce Du, Ji Miao, Feng Wang, Yizhou Wang, Xiaofeng Lu, Wenxian Guan

**Affiliations:** ^1^Department of General Surgery, Affiliated Drum Tower Hospital, Medical School of Nanjing University, Nanjing, China; ^2^Department of General Surgery, Nanjing Drum Tower Hospital Clinical College of Nanjing Medical University, Nanjing, China; ^3^Department of Gastroenterology, Affiliated Drum Tower Hospital, Medical School of Nanjing University, Nanjing, China

**Keywords:** GPER1, EMT, migration, invasion, gastric cancer

## Abstract

G protein coupled estrogen receptor (GPER1) is a membrane estrogen receptor, belonging to the seven-transmembrane G protein-coupled receptors family, and has important biological functions in cancer. However, the functional role of GPER1 in gastric cancer (GC) remain incompletely understood. In the present study, we employed gene set enrichment analysis and discovered that GPER1 expression was concomitant with EMT process and was positively correlated with activation of the PI3K/AKT pathway in GC. Knockdown of GPER1 with siRNA suppressed the proliferation, migration, and invasion of AGS and MGC-803 GC cells. Knockdown of GPER1 also downregulated the mesenchymal markers N-cadherin and vimentin, upregulated E-cadherin, an epithelial marker, and suppressed expression of the Snail, Slug and Twist1 transcription factors, indicating that knockdown of GPER1 inhibited EMT. Moreover, 740Y-P, a PI3K activator, reversed the effects of GPER1 knockdown on EMT processes. Overexpression of GPER1 with plasmid can further prove these findings. In summary, these data demonstrate that GPER1 inhibition suppresses the proliferation, migration, and invasion of gastric cancer cells by inhibiting PI3K/AKT-mediated EMT. Our study elucidated the function of GPER1 in gastric cancer, and we identified PI3K/AKT-mediated EMT as a novel mechanism by which GPER1 contributes to proliferation, migration, and invasion of gastric cancer. These data suggest that combining inhibition of GPER1 and PI3K may be a potential therapeutic approach to inhibit gastric cancer metastasis.

## Introduction

Gastric cancer is the third leading cause of cancer-related death worldwide, and is the third leading cause of cancer mortality in China (Chen et al., 2016; Bray et al., 2018). Gastric cancer (GC) originates from the gastric mucosal epithelium, and while it can occur in any part of the stomach, more than half of GC tumors occur in the gastric antrum (Waldum and Fossmark, 2018). Surgery remains the only curative treatment for gastric cancer, but the overall prognosis is poor; patients with GC have an average 5-years survival rate of <20%, primarily because of late diagnosis due to lack of early clinical symptoms (Correa, 2013). In addition, systemic chemotherapy is also used to treat GC, but the objective response rate of advanced GC to chemotherapy remains unsatisfactory, with a median progression-free survival of 4–7 months and overall survival of 9–14 months (Wang et al., 2016). It is therefore of urgent need to identify novel therapeutic targets that could improve treatment options and outcomes for patients with GC.

GPER1 belongs to the seven-transmembrane G protein-coupled receptors family, which mediate fast signal responses and transcriptional events (Molina et al., 2017). GPER1 is a membrane estrogen receptor that regulates cell growth, migration, apoptotic cell death, and other cancer-related biological functions (Feldman and Limbird, 2017). However, there is contradictory evidence indicating that GPER1 act as either a tumor promoter or a tumor suppressor in different cancer contexts. For example, estradiol (E2) induces proliferation of BPH-10-3 thyroid cancer cells in a concentration and time-dependent manner, resulting in overexpression of phosphorylation AKT/mTOR; these effects were reversed by G15, a GPER1-specific inhibitor (Manfroi et al., 2020). Furthermore, GPER1 expression was found to be higher in HCC tissue than in cancer-adjacent tissue (Chaturantabut et al., 2019). GPER1 mediated the proliferation of hepatocytes via PI3K and mTOR signaling, and chemical inhibition or knockdown of GPER1 significantly reduced tumor growth in a zebrafish model (Chaturantabut et al., 2019). On the contrary, G1, a GPER1 agonist, inhibited the proliferation and apoptosis of A549 lung cancer cells mediated by oxidant and antioxidant molecules (Kurt et al., 2015), and suppressed the migration and angiogenesis of triple negative breast cancer cells through suppressing the NF-κB/IL-6 pathway (Liang et al., 2017). GPER1 expression is associated with poorer prognosis in patients with gastric cancer (Wesolowska et al., 2016), but the specific role of GPER1 in gastric cancer development and progression is not well-understood.

More than 90% of cancer-related death is related to metastasis, and the epithelial-to-mesenchymal transition (EMT) is critically involved in tumor metastasis (Diepenbruck and Christofori, 2016). EMT refers to the transformation of epithelial cells into a mesenchymal state, and is associated not only with tumor metastasis but also with tumor initiation, invasion, and resistance to therapy (Pastushenko and Blanpain, 2019). During EMT, cell morphology, cell-cell adhesion, and many cellular pathways are altered, leading to invasion of cancer cells into the surrounding tissue and eventually to tumor metastasis (Lamouille et al., 2013). The phosphoinositol-3-kinase (PI3K) pathway plays an important role in cell proliferation, migration, and invasion, mediates EMT processes (Wu et al., 2019), and is emerging as a promising target in cancer therapy (Barrett et al., 2012; Sharma et al., 2017; Narayanankutty, 2019). G protein-coupled receptors can directly activate most class I PI3k subunits through interaction with Gβγ protein subunits (Pal and Mandal, 2012). However, whether the GPER1 contributes to gastric cancer progression and metastasis through a mechanism involving the PI3K pathway and EMT remains unclear.

In this study, we used siRNA to knock down the expression of GPER1 in two gastric cancer cell lines, AGS and MGC-803. We evaluated changes in cell proliferation, migration, and invasion after GPER1 knockdown. In addition, we also used the PI3K activator 740Y-P to assessed the role of the PI3K pathway in GPER1-mediated proliferation, migration, and invasion of GC cells. Our findings suggest that GPER1 may represent a novel target in the treatment of gastric cancer.

## Materials and Methods

### Cell Culture

Two human gastric cancer cell lines, AGS and MGC-803, were purchased from the American Type Culture Collection (Manassas, VA, USA). AGS and MGC-803 cells were cultured in RPMI 1640 medium (Gibco, Waltham, MA, USA) supplemented with 10% fetal bovine serum (FBS; Gibco, Waltham, MA, USA) and 1% penicillin/streptomycin (Gibco, Waltham, MA, USA). Culture medium was refreshed and the cells were passaged every 3 days.

### siRNA Transfection Assay

siRNAs specifically targeting GPER1(Shanghai GenePharma Co., Ltd.) were transfected in GC cells using interferin reagent (Polyplus, New York, USA), according to the manufacturer's instructions. Firstly, dilute 5p moles of siRNA in 200 μL of medium without serum. Vortex 20 s and spin down. Then, add 8 μL of interferin reagent. Vortex 20 s, spin down and incubate 10 min at RT. During the incubation time, remove 2 ml of serum containing medium. Finally, add transfection mix to the cells in serum containing medium. The siRNA target sequences were GCACCTTCATGTCGCTCTT. The knockdown efficiency of GPER1 siRNA was examined by western blot.

### Plasmid Transfection Assay

GPER1 plasmid (Shanghai GenePharma Co., Ltd.) were transfected in GC cells using Lipofectamine 2000 reagent (Thermo Fisher Scientific, Waltham, MA, USA), according to the manufacturer's instructions. The overexpression efficiency of GPER1 siRNA was examined by western blot.

### Lentiviral Transduction

The lentiviral vector containing gene-specific shRNAs against GPER1 and a control lentiviral vector encoding scrambled shRNA were purchased from Shanghai Gene-Pharma Co., Ltd (Shanghai, China). Human GC cell lines were transduced with the lentiviral particles along with polybrene and were selected by puromycin (1 mg/mL) (Thermo Fisher Scientific, Waltham, MA, USA) for 2 weeks. The knockdown efficiency was examined by western blot.

### Western Blot Analysis

Cells lysates were collected and 30 mg protein from each sample were separated using 10% SDS-PAGE. The proteins were then electro-transferred to a PVDF membrane (Millipore, Boston, MA, USA). The PVDF membranes were blocked in 10% skimmed milk. Membranes were then incubated with primary antibodies, including anti-GPER1 (Abcam, ab39742, 1:1000 dilution), anti-GAPDH (Abcam, ab181602, 1:10000 dilution), anti-PI3K (Abcam, ab151549, 1:1000 dilution), anti-p-PI3K (Abcam, ab182651, 1:1000 dilution), anti-AKT (Abcam, ab179463, 1:1000 dilution), anti-p-AKT (Abcam, ab81283, 1:1000 dilution), anti-MMP9 (Abcam, ab76003, 1:1000 dilution), anti-MMP2 (Abcam, ab92536, 1:1000 dilution), anti-E-Cadherin (Abcam, ab1416, 1:1000 dilution), anti-N-Cadherin (Abcam, ab18203, 1:1000 dilution), anti-Vimentin (Abcam, ab92547, 1:1000 dilution), anti-snail (Abcam, ab216347, 1:1000 dilution), anti-slug (Abcam, ab27568, 1:1000 dilution), and anti-TWIST1 (Abcam, ab50887, 1:1000 dilution) at 4°C overnight. Membranes were then incubated with horseradish peroxidase-conjugated secondary antibody (Abacm AB6721,1:10000 dilution) at room temperature for 2 h. Chemiluminescent detection reagents (Millipore, Boston, MA, USA) were used to visualize the protein bands.

### Reverse Transcription Quantitative Real-Time Polymerase Chain Reaction (RT-qPCR)

Total RNA was extracted from the GC cells, and was then reverse-transcribed into cDNA using a RT-PCR kit (Takara, Kyoto, Japan). Gene-specific primers were designed using Primer Express version 2.0 software (Applied Biosystems. Inc., Milan, Italy) and are as follows: human GPER1 Forward: 5'-AGTCGGATGTGAGGTTCAG-3′ and Reverse.: 5'-TCTGTGTGAGGAGTGCAAG-3′; human GAPDH Forward: 5'- GGAGTCCACTGGCGTCTTCA-3′ and Reverse.: 5'- GGGGTGCTAAGCAGTTGGTG-3′. The RT-PCR assays were performed using SYBR Green Premix Ex Taq on an ABI ViiA 7DX RT-PCR machine. Human GAPDH was used as an internal reference gene. Relative mRNA expression was calculated according to the 2^−−ΔΔCt^ method.

### Bioinformatics Analysis

We analyzed the relationship of GPER1 expression with gastric cancer prognosis by using Oncolnc (http://www.oncolnc.org) and KM Plotter (http://kmplot.com/analysis/index.php?p=service&cancer=gastric). Gene expression data were acquired from The Cancer Genome Atlas (TCGA; https://portal.gdc.cancer.gov/projects/TCGA-LIHC) and were analyzed using Gene Set Enrichment Analysis (GSEA).

### Cell Viability Assays

The AGS and MGC-803 cell lines were inoculated into 96-well microplates at a density of 5,000 cells per well. After cells had attached for ~24 h, the culture medium was replaced with complete medium containing different concentrations of oxaliplatin for 48 h. Cell proliferation was determined using a cell counting kit-8 (CCK-8) assay (Dojindo, Kumamoto, Japan), according to the manufacturer's instructions. Briefly, 10 μL of CCK-8 working solution per 100 μL medium was added to the microplates and the cells were incubated for 1.5 h. The OD450 value was determined by using a MRX II microplate reader (Dynex, Chantilly, VA, USA).

### EdU Staining

GC cells were incubated with 10 μM Edu (C0071L, Beyotime, Shanghai, P.R. China) for 2 h and were fixed using 4% paraformaldehyde for 15 min at room temperature. Then, GC cells were washed with PBS containing 3% BSA for 3×5 min and then permeabilized with PBS containing 0.3% Triton X-100 for 10–15 min. The cells were washed with PBS containing 3% BSA extensively, and then were incubated with Click Additive Solution for 30 min in dark. Next, the cells were washed with PBS containing 3% BSA for 3 × 5 min and then incubated with Hoechst 33342 for 10 min in dark. Finally cells were washed and observed by Olympus microscope.

### Cell Cycle Analysis

GC cells were stained with propidium iodide (PI; Dawen) and analyzed by flow cytometry. ModFit software (Verity Software House) was used for quantitative analysis of cell cycle. Cell proliferation is expressed as the percentage of S + G2/M phase cells.

### Wound Healing Assay

Cells were inoculated in 6-well plates at a density of 5 × 10^5^/well, and cultured to 90% confluency. A wound track was made on each plate with a plastic scraper and the plates were washed with PBS to remove loose cell fragments. After 48 h of culture, the migration distance was measured using an Olympus microscope and the migration rate was calculated. All experiments were performed for repeated three times.

### Cell Migration Assay

Transwell chambers (Corning) were used to evaluate migration in the Transwell assays. Cells were inoculated in the upper chamber at a density of 5 × 10^4^/chamber, and RPMI-1640 containing 10% fetal bovine serum were added in the lower chamber as chemoattractant. After incubation at 37°C for 24 h, the upper chamber cells were removed with cotton swabs and the cells in the lower chamber were fixed with formaldehyde for 30 min and stained with 0.1% crystal violet for 20 min. All experiments were performed for repeated three times. Ten fields were randomly selected and positively stained cells were counted using an Olympus microscope.

### Cell Invasion Assay

Transwell chambers (6.5 mm, Costar, Corning, NY, USA) were pre-coated with Matrigel for 30 min. Then, 1 ×10^5^ cells suspended in serum-free RPMI 1640 medium were inoculated into the upper chamber, with RPMI 1640 medium containing 10% fetal bovine serum added in the lower chamber. After incubation at 37°C for 24 h, the upper chamber cells were removed with cotton swabs and the cells in the lower chamber cells were fixed with formaldehyde for 30 min and stained with 0.1% crystal violet for 20 min. All experiments were performed for repeated three times. Ten fields were randomly selected and positively stained cells were counted using an Olympus microscope.

### Xenograft and Peritoneal Dissemination Model

Four weeks old female BALB/c nude mice were purchased from Model Animal Research Center of Nanjing University and housed in a pathogen-free environment in the Animal Laboratory Unit of Nanjing University. For the xenograft model, GPER1 knockdown and control GC cells (2 × 10^6^) resuspended in 200 μL of PBS were injected in the flank of nude mice (5 mice/group). These mice were sacrificed 21 days after tumor cell implantation. At autopsy the tumor was removed and weighed. For peritoneal dissemination model, GPER1 knockdown and control GC cells (3 × 10^6^) in 400 μL PBS were injected into the peritoneal cavity. Peritoneum metastasis was examined and recorded when mice were killed at 14 days after injection.

### Statistical Analysis

SPSS 22.0 (IBM) was used for statistical analysis. The results of three or more independent experiments were expressed as mean ± standard deviation. Comparisons between two groups were performed using the Student's *t*-test. Comparisons between three groups were performed by one-way ANOVA. The chi-square test was used to compare count data. The correlation between two variables was analyzed by linear regression. A difference was considered statistically significant at a *P*-value of *P* < 0.05.

## Results

### Correlation Between GPER1 Expression and Prognosis in Gastric Cancer and Construction of Cell Lines

**Figure 1:**
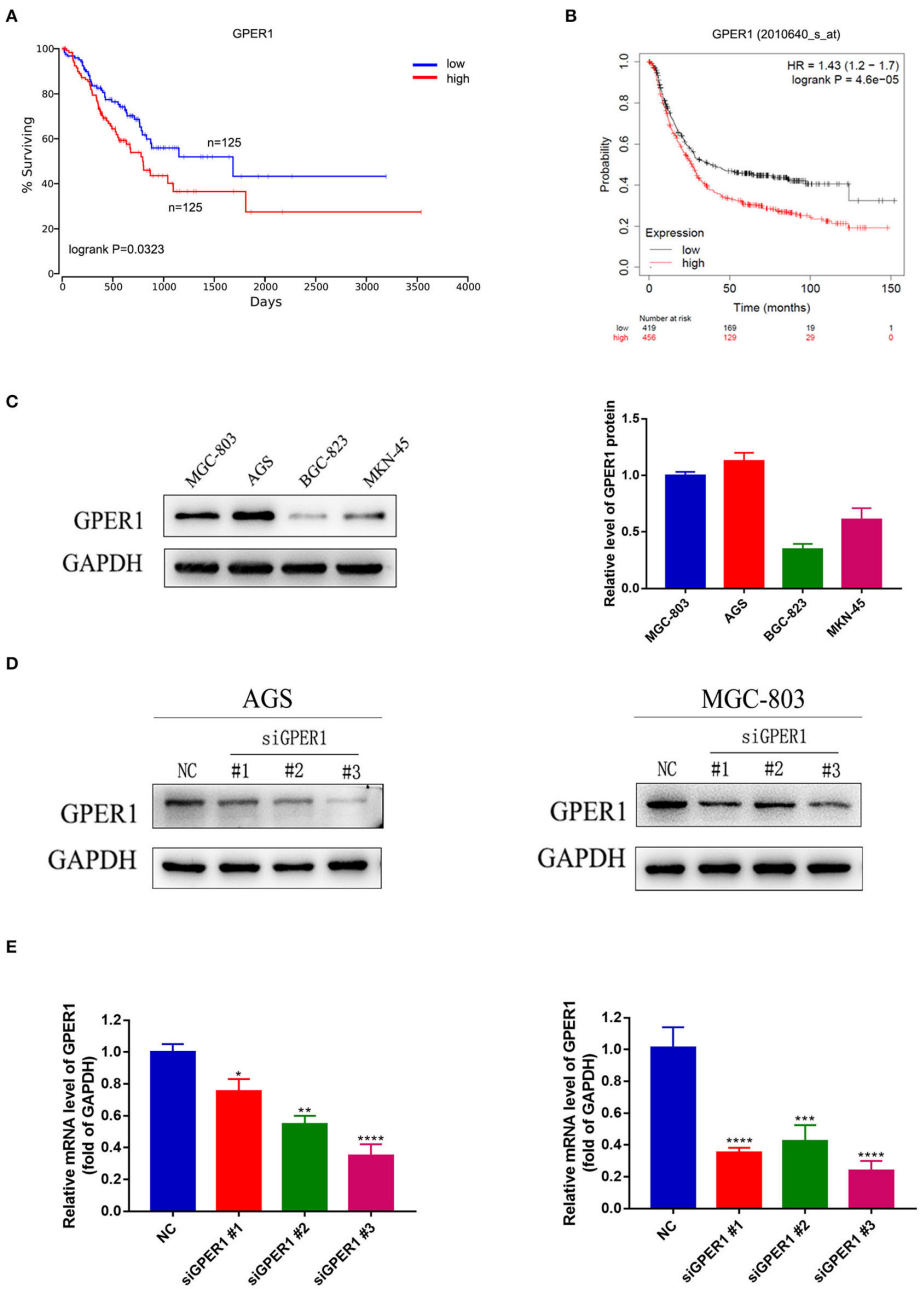
Correlation between GPER1 expression and prognosis in gastric cancer, GPER1 expression in different gastric cancer cell lines, and verification of transfection efficiency. **(A,B)** Correlation of GPER1 expression in gastric cancer patients with overall survival rate; **(C)** Western blot of GPER1 protein and gene expression in four gastric cancer cell lines; GAPDH was used as a loading control; **(D,E)** Protein and gene expression levels of GPER1 in AGS and MGC-802 cells transfected with siRNA targeting GPER1. Results were shown as mean ± SD of three independent experiments, each experiment was performed in triplicate. *P < 0.05; **P < 0.01; ***P < 0.001; ****P < 0.0001.

In the process of seeking the potential value of GPER1 as a therapeutic target in gastric cancer. We used published datasets from the Oncolnc (Figure 1A) and KM plotter (Figure 1B) database and found that gastric cancer patients with high GPER1 expression had significantly worse overall survival than patients with low GPER1 expression. Then we examined the expression of GPER1 by western blot in four gastric cancer cell lines, including MGC-803, AGS, BGC-823, and MKN-45 and found that GPER1 was highly expressed in the AGS and MGC-803 cell lines (Figure 1C). Thus, AGS and MGC-803 cells were selected to evaluate the efficiency of siRNA-mediated GPER1 knockdown. The transfection efficiency was confirmed by western blot and quantitative real-time PCR (Figures 1D,E). The 3^#^ siRNA was found to deliver the most effective knockdown, and was used to conduct subsequent GPER1 knockdown assays.

### GPER1 Is a Regulator of the PI3K/AKT Pathway

**Figure 2:**
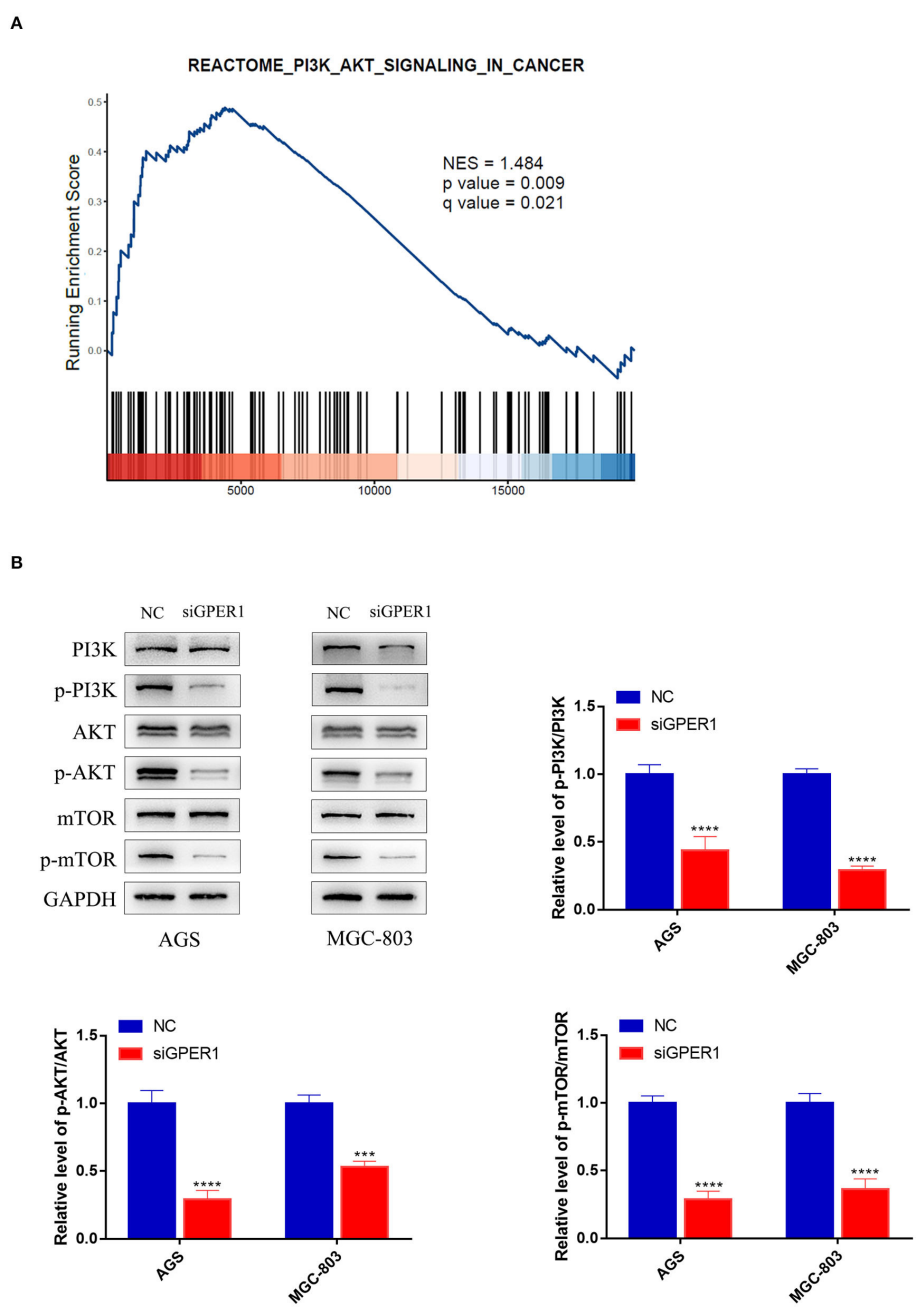
GPER1 is a regulator of the PI3K/AKT pathway. **(A)** Gene Set Enrichment Analysis evaluating GPER1 expression and the PI3K/AKT_SIGNALING pathway in gastric cancer; **(B)** Western blot analysis of p-PI3K, PI3K, p-AKT, AKT, p-mTOR, and mTOR protein expression in AGS and MGC-803 cells transfected with GPER1 siRNA. Results were shown as mean ± SD of three independent experiments, each experiment was performed in triplicate. ***P < 0.001; ****P < 0.0001.

Gene Set Enrichment Analysis was used to analyze the correlation between GPER1 expression in gastric cancer and activation of the PI3K/AKT pathway. A significant positive association was found between GPER1 expression and activation of the PI3K/AKT pathway (Figure 2A). Subsequently, we examined the protein expression levels of phospho-PI3K, PI3K, phospho-AKT, and AKT in the AGS and MGC-803 cell lines with and without GPER1 siRNA transfection (Figure 2B). Western blot analysis demonstrated that p-PI3K and p-AKT were significantly decreased in AGS and MGC-803 cells transfected GPER1 siRNA.

### Knockdown of GPER1 Inhibits the Proliferation of Gastric Cancer Cells

**Figure 3:**
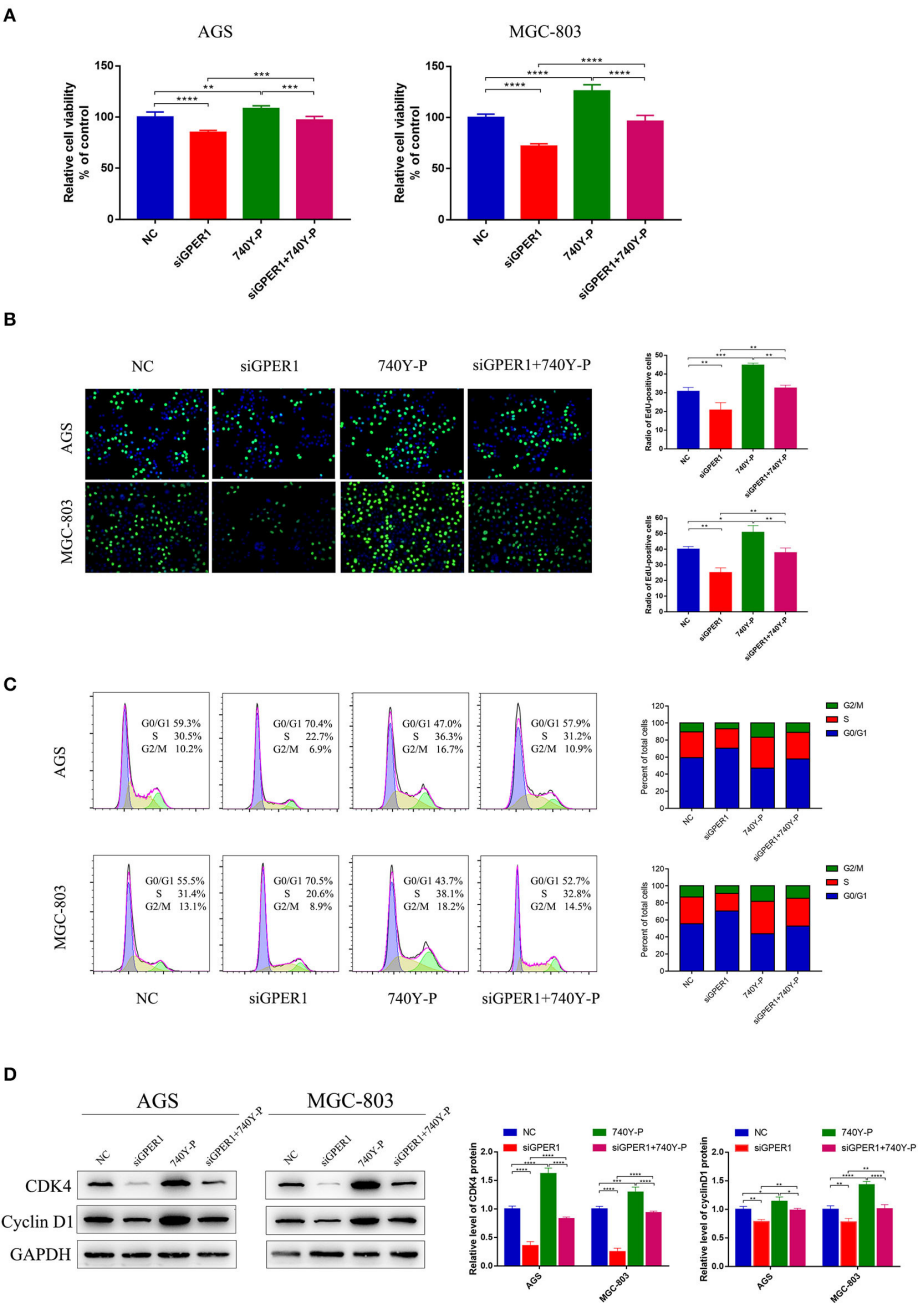
GPER1 knockdown impairs the proliferation of AGS and MGC-803 cells. **(A)** CCK-8 and **(B)** Edu assays were used to evaluate the proliferation of AGS and MGC-803 cells after transfection with siGPER1 for 48 h; **(C)** Flow cytometry and **(D)** CDK4 and cyclin D1 protein levels were used to analyze the cell cycle of AGS and MGC-803 cells after transfection with siGPER1 for 48 h. Control: cells without transfection; GPER1, cells transfected with GPER1 siRNA; 740Y-P, cells treated with 740Y-P; GPER1 + 740Y-P, cells transfected GPER1 siRNA and then treated with 740Y-P. Results were shown as mean ± SD of three independent experiments, each experiment was performed in triplicate. *P < 0.05; **P < 0.01; ***P < 0.001; ****P < 0.0001.

We evaluated the effects of GPER1 knockdown and PI3K pathway activation by 740Y-P, a PI3K activator, on the proliferation of AGS and MGC-803 cells using a CCK-8 assay. The viability of AGS and MGC-803 cells was significantly decreased in the siGPER1 group compared to the control group, and was significantly increased in the 740Y-P group (Figure 3A). Furthermore, the proliferation of cells in the siGPER1+740Y-P group was similar to the control group, indicating that activation of the PI3K/AKT pathway with 740Y-P reversed the anti-proliferative effects of GPER1 knockdown. An Edu thymidine analog incorporation assay was also used to evaluate cell proliferation. The expression of Azide488 (an Edu probe) was highest in the 740Y-P group and lowest in the GPER1 siRNA group (Figure 3B), consistent with the results of CCK-8 assay. The percentage of AGS and MGC-803 cells in the G2/M phase was significantly higher in the GPER1 siRNA group and significantly lower in the 740Y-P group (Figure 3C). The intervention of 740Y-P significantly reversed the impact of GPER1 knockdown on cell cycle arrest in AGS and MGC-803 cells. The same changes were also observed in protein levels of cyclin D1 and CDK4 (Figure 3D), further supporting this conclusion.

### Knockdown of GPER1 Inhibits the Migration and Invasion of Gastric Cancer Cells

**Figure 4:**
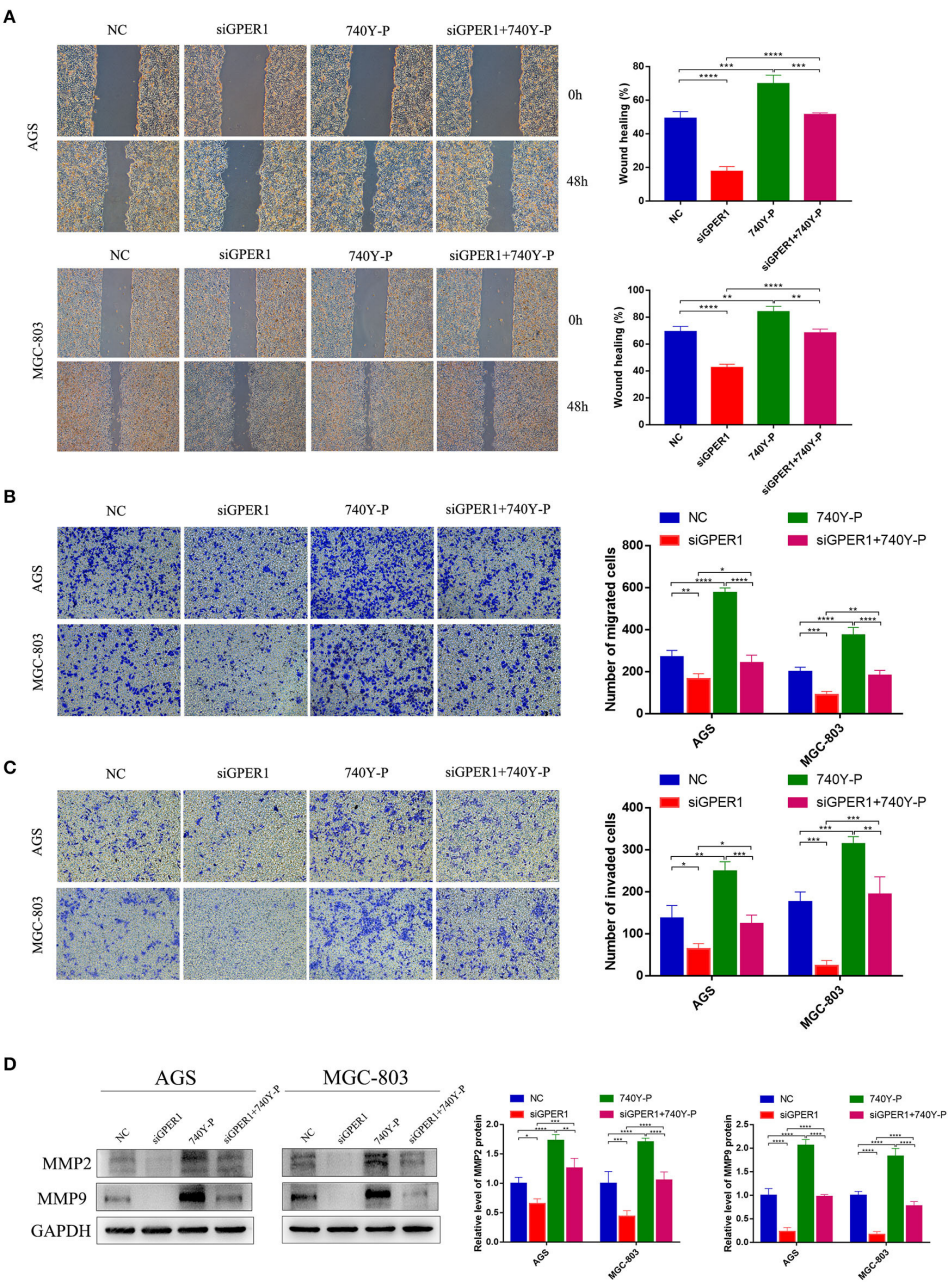
GPER1 knockdown impairs the migration and invasion of AGS and MGC-803 cells. **(A)** Cell mobility was detected by wound healing assay; **(B)** cell migration and **(C)** invasion were detected by transwell assays; **(D)**. Western blot analysis of matrix metalloproteinase 9 (MMP9) and MMP2 protein expression. GAPDH was used as a loading control. Control, cells without transfection; GPER1, cells transfected with GPER1 siRNA; 740Y-P, cells treated with 740Y-P; GPER1 + 740Y-P, cells transfected GPER1 siRNA and then treated with 740Y-P. Results were shown as mean ± SD of three independent experiments, each experiment was performed in triplicate. *P < 0.05; **P < 0.01; ***P < 0.001; ****P < 0.0001.

The migration of AGS and MGC-803 cells was evaluated using wound healing and transwell migration assays. GPER1 knockdown inhibited the migration rates of AGS and MGC-803 cells, while 740Y-P reversed the anti-migration effects of GPER1 knockdown, consistent with changes in the expression of MMP2 and MMP9 (Figures 4A,B,D). Additionally, transwell assays demonstrated that the invasion rates of AGS and MGC-803 cells were significantly decreased in the GPER1 siRNA group and increased in the 740Y-P group, compared to control group (Figure 4C). Changes in the invasion rate ware also correlated with changes in MMP2 and MMP9 expression (Figure 4D).

### Knockdown of GPER1 Downregulates EMT in Gastric Cancer Cells

**Figure 5:**
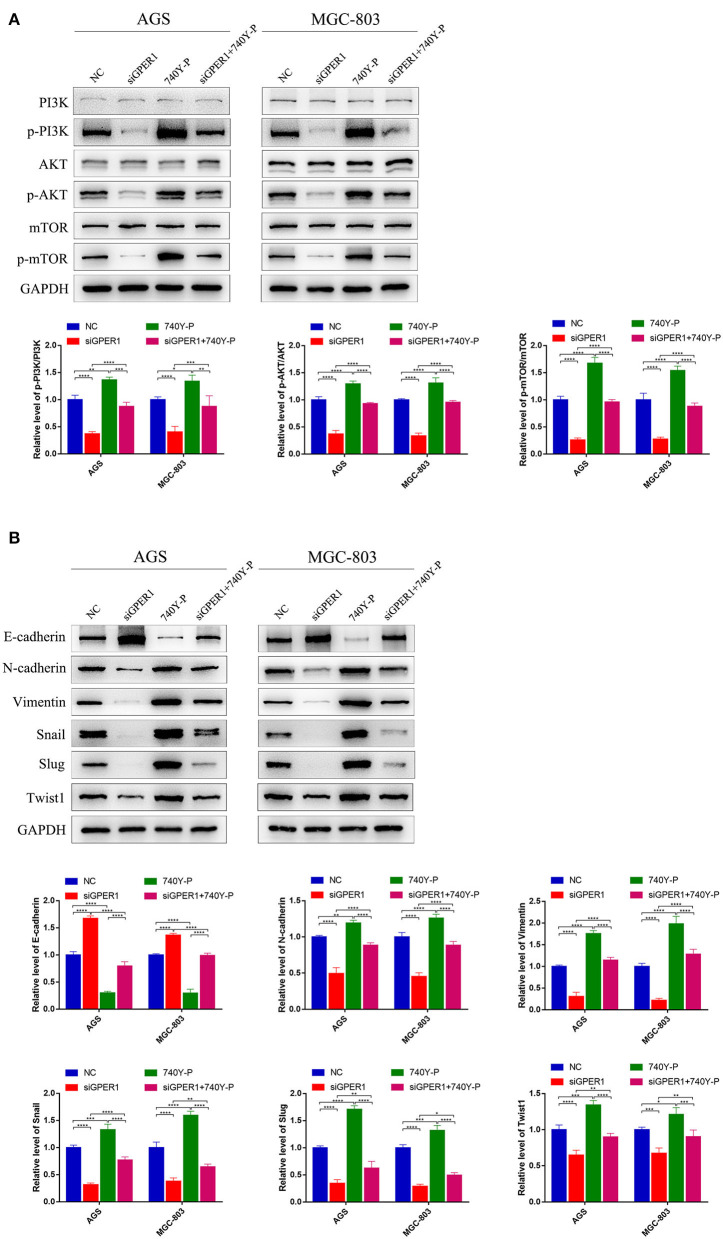
Protein levels of PI3K/AKT and EMT markers in AGS and MGC-803 cells treated with GPER1 siRNA. **(A)** PI3K/AKT pathway and **(B)** EMT markers were detected by western blot, GAPDH was evaluated as an internal control. Control, cells without transfection; GPER1, cells transfected with GPER1 siRNA; 740Y-P, cells treated with 740Y-P; GPER1 + 740Y-P, cells transfected GPER1 siRNA and then treated with 740Y-P. Results were shown as mean ± SD of three independent experiments, each experiment was performed in triplicate. *P < 0.05; **P < 0.01; ***P < 0.001; ****P < 0.0001.

The levels of EMT markers were evaluated by western blot. After transfection with GPER1 siRNA, E-cadherin, a marker of epithelial cells, was upregulated, and N-cadherin and vimentin, markers of mesenchymal cells, were downregulated in AGS and MGC-803 cells (Figure 5A). Moreover, the changes in these EMT markers were reversed by treatment with 740Y-P (Figure 5B). Additionally, 740Y-P induced upregulation of Snail, Slug and Twist1, key transcription factors of EMT, which were repressed by GPER1 siRNA. These data confirm the essential role of GPER1 in promoting EMT of gastric cancer cells via activation of the PI3K/AKT signaling pathway.

### Overexpression of GPER1 Promotes Proliferation, Migration, and Invasion of Gastric Cancer Cells Through PI3K/AKT–Mediated EMT

**Figure 6:**
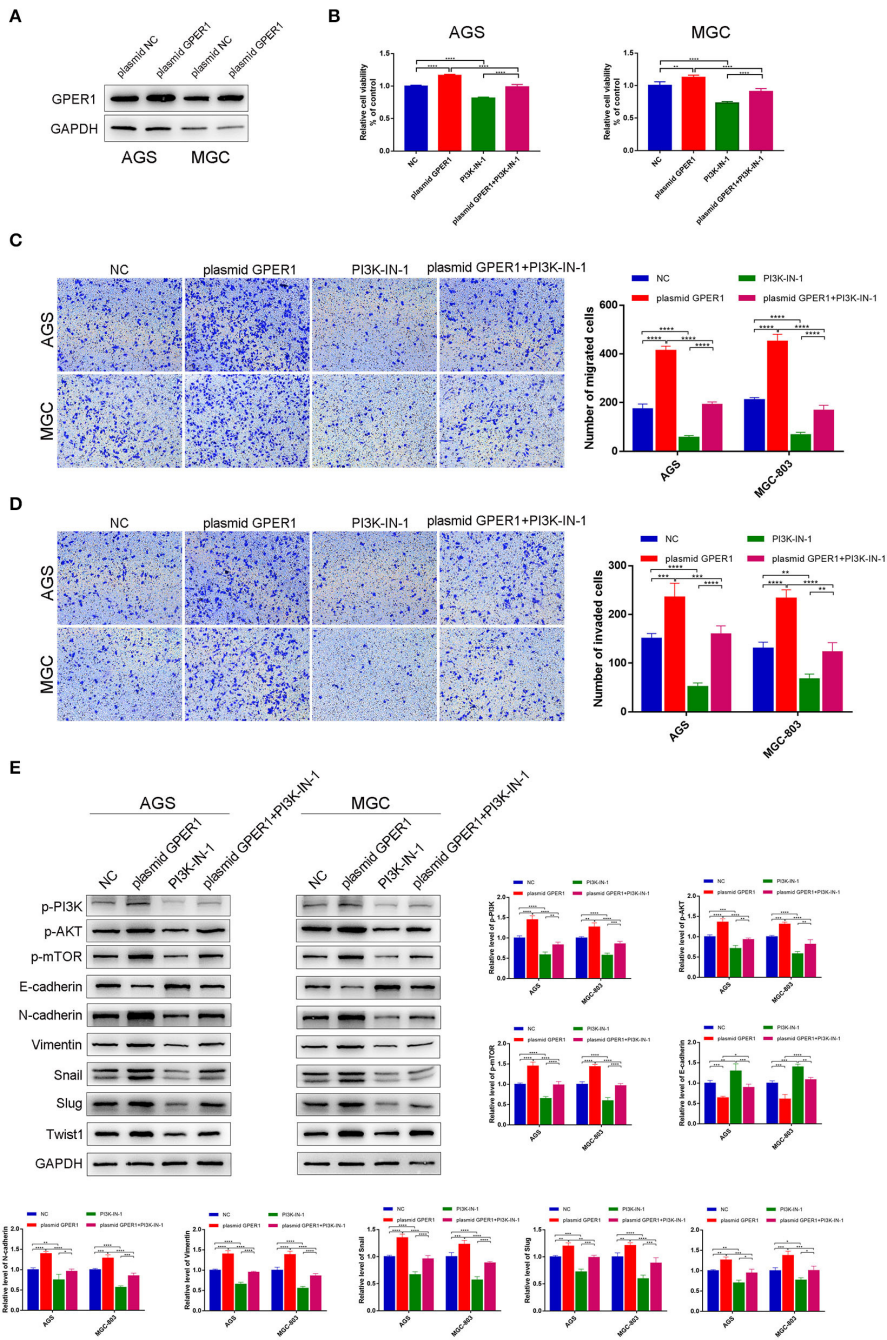
Overexpression of GPER1 promotes proliferation, migration, and invasion of gastric cancer cells. **(A)** Protein levels of GPER1 in AGS and MGC-802 cells transfected with plasmid targeting GPER1; **(B)** CCK-8 was used to evaluate the proliferation of AGS and MGC-803 cells after transfection with GPER1 plasmid for 48 h; **(C)** cell migration and **(D)** invasion were detected by transwell assays; **(E)**. Western blot analysis of PI3k pathway and EMT markers were detected. *P < 0.05; **P < 0.01; ***P < 0.001; ****P < 0.0001.

To further confirm the effects of GPER1 on proliferation, migration and invasion of GC cell lines, we have instead overexpress the GPER1 in AGS and MGC-803 using plasmid targeting GPER1 (Figure 6A). The viability of AGS and MGC-803 cells was significantly increased in the plasmid GPER1 group compared to the control group and PI3K-IN-1, a PI3K/AKT inhibitor, can reverse the effect of GPER1 on the proliferation (Figure 6B). Additionally, overexpression of GPER1 promoted the migration and invasion rates of AGS and MGC-803 cells while PI3K-IN-1 reversed these effects (Figures 6C,D). What's more, E-cadherin expression level was decreased and expression levels of N-cadherin, Vimentin, Snail, Slug, and Twist1 were increased in GPER1 overexpressed AGS and MGC-803 cell line (Figure 6E), which trends are also opposite to those observed in GPER1 knockdown GC cell lines. These opposite trends observed in GPER1 overexpression compared to GPER1 knockdown further convinced the relationship between GPER1 expression and GC cell line proliferation, migration and invasion.

### Knockdown of GPER1 Inhibits Tumor Growth and Peritoneal Dissemination *in vivo*

**Figure 7:**
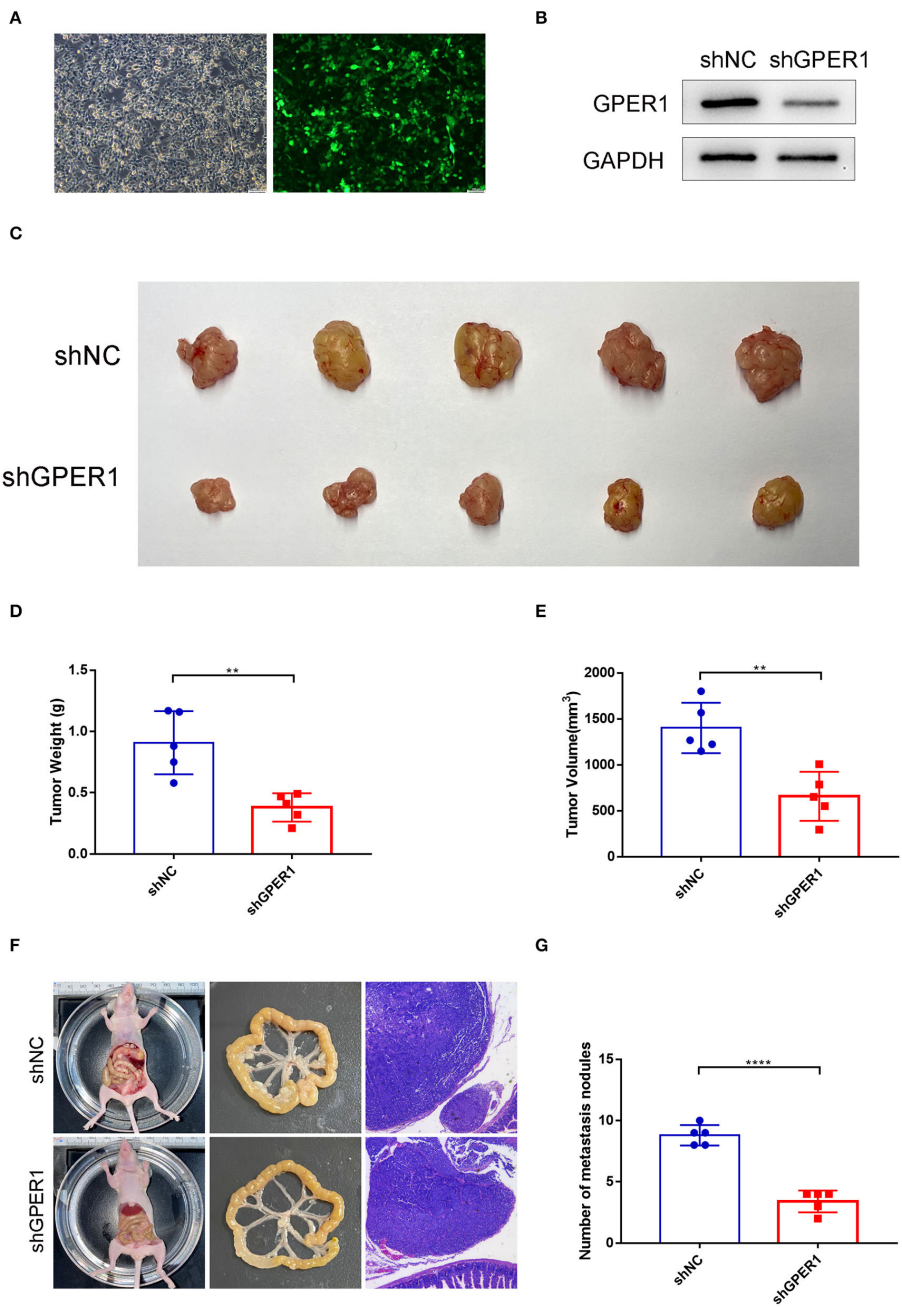
GPER1 knockdown inhibited tumor growth and peritoneal dissemination *in vivo*. **(A)** Fluorescence was used to verify the infection efficiency (magnification ×200); **(B)** Western blot analysis of knockdown efficiency of shGPER1; **(C)** Image of tumors of mice in shGPER1 and shNC groups; **(D)** Volume of tumor of mice in in shGPER1 and shNC groups; **(E)** Weight of tumor of mice in shGPER1 and shNC groups; **(F)** Representative intestines and H&E staining of scattered tumors in nude mice; **(G)** The quantification of metastases in nude mice was counted. **P < 0.01; ****P < 0.0001.

To determine the effects of GPER1 *in vivo*, xenograft and peritoneal dissemination model were established. AGS and MGC-803 cells were infected with lentivirus expressing shRNA-GPER1 or shRNA-NC. The efficiency of infection and knockdown were verified by fluorescence and western blot (Figures 7A,B). For xenograft model, 21 days after subcutaneous injection, mice were sacrificed, and the weight and volume of tumors were measured. The weight and volume of tumors in GPER1 knockdown group were decreased compared to control group (Figures 7C–E). For peritoneal dissemination model, 14 days after peritoneal cavities injection, mice were sacrificed. The results showed that knockdown of GPER1 reduced the mesenteric metastatic nodules in the intestinal wall of nude mice (Figures 7F,G).

## Discussion

GPER1 is a specific estrogen receptor that is independent of the classical estrogen receptors, such as ERα and ERβ. About 20% of cancers exhibit alterations in GPER1 (Filardo, 2018). However, GPER1 has contradictory roles as either a tumor-suppressor or a tumor-promoter in, depending on the specific cancer context. GPER1 acts as a tumor promoter in thyroid cancer (Manfroi et al., 2020), hepatocellular carcinoma (Chaturantabut et al., 2019), and renal cell adenocarcinoma (Feldman et al., 2016), whereas GPER1 can inhibit the proliferation of lung cancer (Narayanankutty, 2019), prostate cancer (Salata et al., 2019), and estrogen receptor-negative breast cancer cells (Wei et al., 2014). The roles of GPER1 in gastric cancer (GC) have not been fully clarified. In this study, we evaluated the effects of GPER1 on AGS and MGC-803 GC cells through siRNA knockdown of GPER1.

Our data demonstrate that knockdown of GPER1 suppressed the proliferation of gastric cancer cell lines. The Edu assays showed that the proliferation of AGS and MGC-903 cells was significantly decreased by the GPER1 knockdown, which is consistent with studies in ovarian cancer (Ignatov et al., 2013). In addition, we also found that GPER1 knockdown significantly decreased the number of cells in G2/M phase and increased the number of cells in the G0/G1 phase, in accordance with changes in CDK4 and cyclin D1 expression. These results indicate that knockdown of GPER1 inhibited the proliferation of gastric cancer cells by inducing the cell cycle arrest in the G0/G1 phase. What's more, GPER1 overexpression could promote cell proliferation and GPER1 knockdown could inhibited tumor growth *in vivo*. Another estrogen receptor, ERα has shown similar functions on cell proliferation as GPER1 (Wang et al., 2014). A meta-analysis which analyzed studies and The Cancer Genome Atlas (TCGA) data of ERs expression showed that ERα expression may be associated with poor prognosis in GC patients and ERβ was negatively associated with lymph node metastasis (Ge et al., 2018). These findings indicated the importance of estrogen receptors in the progression of GC. However, the underlying signaling pathways are still largely unknown and whether these factors have any interactions requires further investigation.

EMT is a biological process whereby epithelial tumor cells lose epithelial features and acquire a mesenchymal phenotype (Thiery et al., 2009). EMT is an important biological phenomenon through which cancer cells can acquire enhanced migratory and invasive abilities, resulting in the progression of cancer metastasis (Savagner, 2010; Li et al., 2019). EMT is involves a shift from expression of the epithelial marker, E-cadherin, to favor expression of mesenchymal markers, including N-cadherin and vimentin; these changes occur at the transcriptional level through the activity of EMT transcription factors, such as Snail, Slug, and Twist1 (Nieto et al., 2016; Zhang et al., 2019). EMT is also closely related to the invasion and metastasis of gastric cancer (Dong et al., 2017). In the current study, we found that silencing of GPER1 inhibited the EMT by upregulating E-cadherin and downregulating N-cadherin and vimentin, resulting in a decline in the migration and invasion of gastric cancer cell lines. Silencing of GPER1 also reduced the expression of Snail, Slug and Twist1. GPER1 overexpression could promote the progression of EMT and promote GC cell migration and invasion. Moreover, GPER1 knockdown could inhibit peritoneal dissemination *in vivo*. These results indicated that GPER1 knockdown reduced gastric cancer migration and invasion through inhibition of EMT. EMT is critical in the progression of GC and was linked to advanced GC stage and poor prognosis of GC patients.

GSEA analysis indicated that GPER1 expression is positively correlated with the PI3K/AKT pathway. The PI3K/AKT pathway regulates EMT (Ge et al., 2018), and previous studies demonstrated that the PI3K/AKT pathway plays an important role in the migration and invasion of gastric cancer (Ye et al., 2012). Therefore, we further investigated the potential for the PI3K/AKT pathway to be involved in GPER1-mediated EMT of GC cells. We showed that knockdown of GPER1 downregulated the PI3K/AKT pathway, thereby inhibiting EMT and suppressing the migration and invasion of gastric cancer cells. The PI3K activator, 740Y-P, could partially reverse the effects of GPER1 knockdown on the migration and invasion of gastric cancer cells. These data further demonstrated the contribution of GPER1, the PI3K/AKT pathway, and EMT to these proliferation, migration, and invasion of gastric cancer, which is consistent with previous reports (Sun et al., 2018). What's more, GPER1 has been reported to induce robust EMT in cancer cells (De Francesco et al., 2018), further supporting the conclusion that GPER1 plays an important role in the EMT of gastric cancer cells. These results indicate that GPER1 knockdown suppresses the EMT in gastric cancer through inhibition of the PI3K/AKT pathway.

In conclusion, our study demonstrated that knockdown of GPER1 blocked EMT in gastric cancer cells by inhibiting the PI3K/AKT pathway, leading to inhibition of proliferation, migration, and invasion of gastric cancer cells. The combined targeting of GPER1 and the PI3K/AKT pathway may serve as a novel therapeutic strategy for gastric cancer. However, this study was limited to *in vitro* assessments, and the effects of GPER1 inhibition in preclinical models need to be further evaluated.

## Data Availability Statement

The original contributions presented in the study are included in the article/supplementary materials, further inquiries can be directed to the corresponding author/s.

## Ethics Statement

The animal study was reviewed and approved by Nanjing Drum Tower Hospital.

## Author Contributions

EX and XX analyzed and interpreted data and drafted the manuscript. CJ made acquisition of data and performed statistical analysis. ZL, ZY, CZ, XW, SD, JM, FW, and YW participated in studies selection, data extraction, and provided statistical expertise. XX, XL, and WG conceived of the study, participated in its design, and analyzed and interpreted the data. All authors read and approved the final manuscript.

## Conflict of Interest

The authors declare that the research was conducted in the absence of any commercial or financial relationships that could be construed as a potential conflict of interest.
