# Corrigendum: GPER1 Silencing Suppresses the Proliferation, Migration, and Invasion of Gastric Cancer Cells by Inhibiting PI3K/AKT–Mediated EMT

**DOI:** 10.3389/fcell.2022.841792

**Published:** 2022-02-23

**Authors:** En Xu, Xuefeng Xia, Chaoyu Jiang, Zijian Li, Zhi Yang, Chang Zheng, Xingzhou Wang, Shangce Du, Ji Miao, Feng Wang, Yizhou Wang, Xiaofeng Lu, Wenxian Guan

**Affiliations:** ^1^ Department of General Surgery, Affiliated Drum Tower Hospital, Medical School of Nanjing University, Nanjing, China; ^2^ Department of General Surgery, Nanjing Drum Tower Hospital Clinical College of Nanjing Medical University, Nanjing, China; ^3^ Department of Gastroenterology, Affiliated Drum Tower Hospital, Medical School of Nanjing University, Nanjing, China

**Keywords:** GPER1, EMT, migration, invasion, gastric cancer

Error in Figure

In the original article, there was a mistake in Figures 3, 4 as published. In Figures 3D, 4C, we put the wrong picture due to carelessness. The corrected Figures 3, 4 appear below.

**FIGURE 3:**
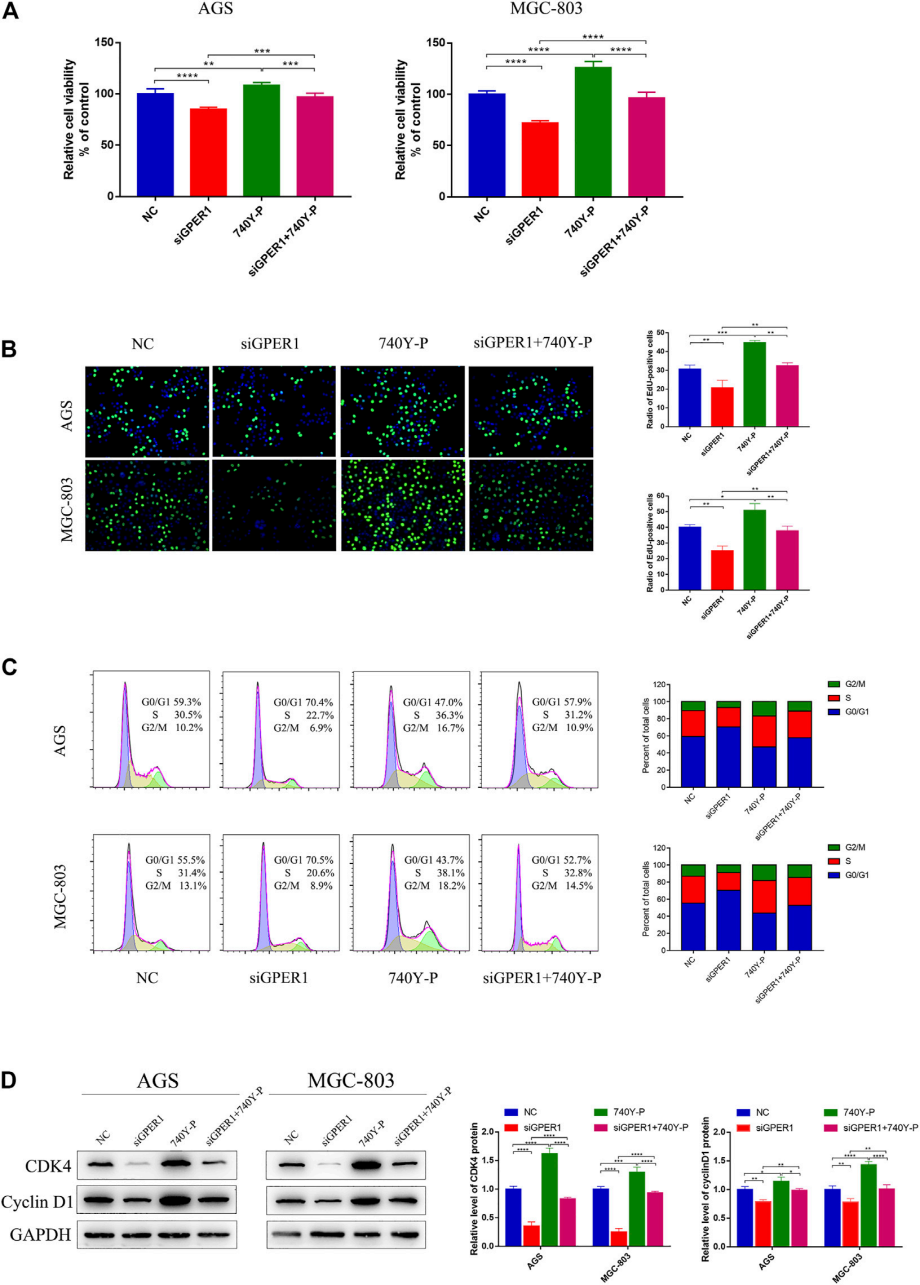
GPER1 knockdown impairs the proliferation of AGS and MGC-803 cells. **(A)** CCK-8 and **(B)** Edu assays were used to evaluate the proliferation of AGS and MGC-803 cells after transfection with siGPER1 for 48 h; **(C)** Flow cytometry and **(D)** CDK4 and cyclin D1 protein levels were used to analyze the cell cycle of AGS and MGC-803 cells after transfection with siGPER1 for 48 h. Control: cells without transfection; GPER1, cells transfected with GPER1 siRNA; 740Y-P, cells treated with 740Y-P; GPER1 + 740Y-P, cells transfected GPER1 siRNA and then treated with 740Y-P. Results were shown as mean ± SD of three independent experiments, each experiment was performed in triplicate. *p < 0.05; **p < 0.01; ***p < 0.001; ****p < 0.0001.

**FIGURE 4:**
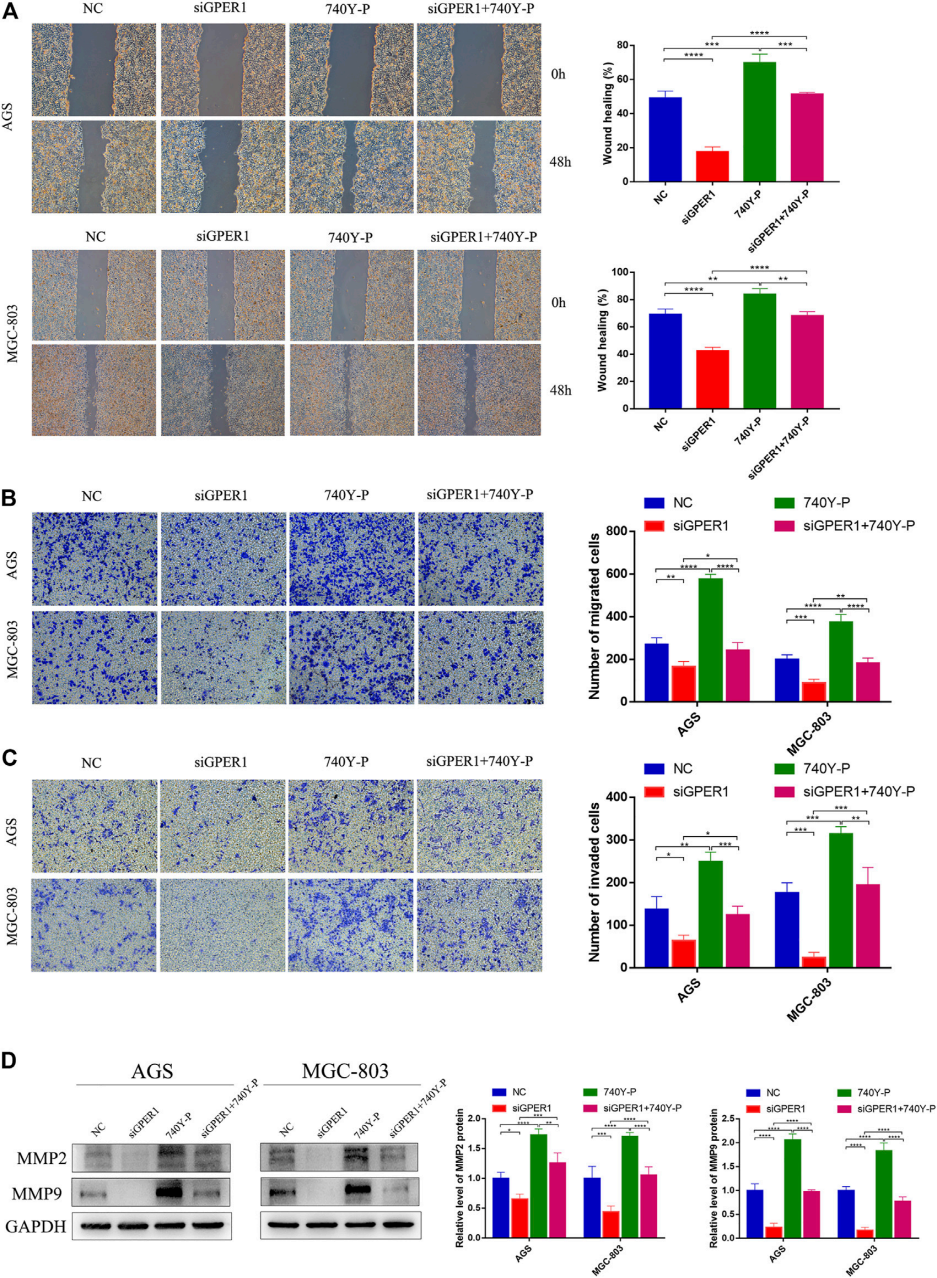
GPER1 knockdown impairs the migration and invasion of AGS and MGC-803 cells. **(A)** Cell mobility was detected by wound healing assay; **(B)** cell migration and **(C)** invasion were detected by transwell assays; **(D)**. Western blot analysis of matrix metalloproteinase 9 (MMP9) and MMP2 protein expression. GAPDH was used as a loading control. Control, cells without transfection; GPER1, cells transfected with GPER1 siRNA; 740Y-P, cells treated with 740Y-P; GPER1 + 740Y-P, cells transfected GPER1 siRNA and then treated with 740Y-P. Results were shown as mean ± SD of three independent experiments, each experiment was performed in triplicate. *p < 0.05; **p < 0.01; ***p < 0.001; ****p < 0.0001.

The authors apologize for this error and state that this does not change the scientific conclusions of the article in any way. The original article has been updated.

